# PAK1 confers chemoresistance and poor outcome in non-small cell lung cancer via β-catenin-mediated stemness

**DOI:** 10.1038/srep34933

**Published:** 2016-10-07

**Authors:** Ming-Jenn Chen, De-Wei Wu, Yao-Chen Wang, Chi-Yi Chen, Huei Lee

**Affiliations:** 1 Department of Surgery, Chi Mei Medical Center, Tainan, Taiwan; 2 Graduate Institute of Cancer Biology and Drug Discovery, Taipei Medical University, Taipei, Taiwan; 3 Department of Internal Medicine, Chung Shan Medical University, Taichung, Taiwan; 4 Department of Surgery, Chung Shan Medical University, Taichung, Taiwan

## Abstract

PAK1 confers resistance to the estrogen antagonist tamoxifen in breast cancer. However, a role for PAK1 remains to be elucidated for chemoresistance and prognosis in non-small cell lung cancer (NSCLC). We provide evidence that PAK1 confers cisplatin resistance by increasing β-catenin expression through ERK/GSK3β signaling. The increased β-catenin expression promotes sphere cell formation and expression of stemness markers and this β-catenin-induced stemness is responsible for PAK1-mediated cisplatin resistance. We enrolled 87 NSCLC patients who had received cisplatin-based chemotherapy to confirm the association between PAK1 expression and response to chemotherapy and outcomes. PAK1 expression, evaluated by immunohistochemistry, was positively correlated with pERK and β-catenin expression in lung tumors. Patients with high-PAK1, high-pERK, and high-nuclear β-catenin tumors more frequently showed an unfavorable response to cisplatin-based chemotherapy when compared to their counterparts. Kaplan-Meier and Cox regression analysis also indicated a poorer overall survival (OS) and relapse free survival (RFS) in patients with high-PAK1, high-pERK, and high-nuclear β-catenin tumors. In conclusion, PAK1 confers cisplatin resistance in NSCLC via β-catenin-mediated stemness. Therefore, we suggest that clinical use of a combination of the MEK/ERK inhibitor AZD6244 and cisplatin might improve sensitivity to cisplatin-based chemotherapy and outcomes in NSCLC patients who harbor high-PAK1-expressing tumors.

P21 activated kinase 1 (PAK1) plays a central role in the malignant transformation induced by Ras, Rac, and Cdc42^1^. Activated PAK1 phosphorylates Raf, which, in turn, enhances Ras/Raf/MAPK signaling^2^. PAK1 also interacts with β-catenin to promote β-catenin/TCF4 activation in gastric epithelial cells and it phosphorylates β-catenin at Serine 675, which increases the stability and transcriptional activity of β-catenin in colon cancer cells^3^. In addition, PAK1 is required for colon cancer cell growth and metastasis *in vivo*^4^. Therefore, PAK1 may promote tumor malignancy by activating Ras/Raf/MARK and β-catenin/TCF4 signaling.

Studies that target PAK1 have shown that suppression of its function or expression induces apoptosis of tumor cells^5^,^6^,^7^. Therefore, PAK1 expression may be responsible for the drug resistance observed in various cancers^5^,^8^,^9^,^10^,^11^. For example, PAK1 overexpression promotes resistance to the antiestrogen tamoxifen in breast cancer^10^, resistance to PI3K inhibitors in lymphomas^8^, resistance to the receptor tyrosine kinase inhibitor sunitinib in renal cell carcinoma^9^, and resistance to a MET inhibitor in pancreatic cancer^11^. Recently, PAK1 phosphorylation was confirmed to confer radio-resistance in lung cancer cells^12^. However, a role for PAK1 in the chemoresistance observed in non-small cell lung cancer (NSCLC) remains to be elucidated. In the present study, we have used a cell model and human tissues to demonstrate that PAK1 confers cisplatin resistance in NSCLC cells via a mechanism involving the ERK/GSK3β/β-catenin cascade and β-catenin-mediated stemness.

## Results

### PAK1 expression may be associated with cisplatin resistance

**Figure 1:**
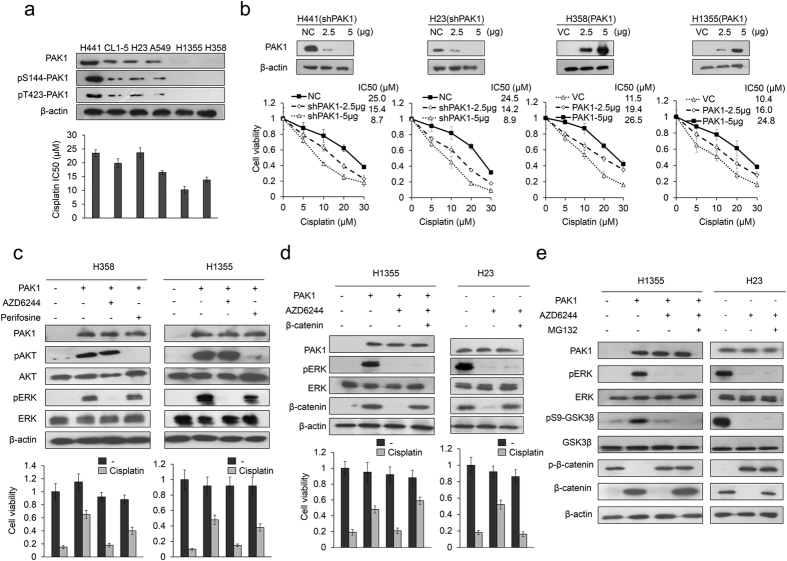
MEK/ERK signaling plays a more important role thanPI3K/AKT signaling in mediating PAK1-mediated cisplatin resistance. (**a**) Six lung cancer cell types were treated with four concentrations of cisplatin and the dose response curves were used to calculate the 50% inhibition concentration (IC50) for cisplatin. The expression of PAK1 and its active forms (pS144-PAK1 and pT423-PAK1) in each lung cancer cell type were examined by western blotting. (**b**) Increasing amounts of PAK1 knockdown plasmid were transfected into high-PAK1 expressing (H441 and H23) cell lines. Alternatively, increasing amounts of expression plasmid were transfected into low PAK1 expressing (H358 and H1355) cell lines. The total amount of transfected DNA was kept constant by adding the control vector. After 48 hr, cell lysates were harvested and evaluated by Western blotting for levels of PAK1 and β-actin protein. β-actin was used as a protein loading control. NC: non-specific shRNA control. VC: Vector control. PAK1-knockdown or PAK1-overexpressing lung cancer cells were treated with four doses of cisplatin and the dose response curves were used to calculate the 50% inhibition concentration (IC50). (**c**) PAK1-overexpressing H358 and H1355 cells were treated for 5 h with inhibitors of PI3K/AKT (10 μM perifosine) and ERK (10 μM AZD6244). The inhibitors were then removed and the cells were treated with 25 μM cisplatin for an additional 48 h. Cell viability was evaluated with the MTT assay. (**d**) H23 and PAK1-overexpressing H1355 cells were transfected with β-catenin overexpression plasmid for 24 h. The cells were then treated with AZD6244 for 5 h, the inhibitor was removed, and the cells were treated with 25 μM cisplatin for an additional 48 h. Cell viability was evaluated with the MTT assay. (**e**) H23 and PAK1-overexpressing H1355 cells were treated with AZD6244 for 5 h, followed by treatment with MG132 for an additional 5 h, and then the cell lysates were evaluated for protein expression by western blotting. All experiments were performed three independent times. The mean values and the standard deviations are indicated as columns with error *bars*. The samples were derived from the same experiment and gels/blots were processed in parallel. Full-length blots are presented in Supplementary Figures S2 and S3.

Six NSCLC cell lines were enrolled to examine whether PAK1 expression levels could be associated with cisplatin resistance. The cisplatin concentration giving 50% cell viability (IC50) in the different cell types was determined using the MTT assay. Evaluation of PAK1 expression by western blotting indicated a generally positive association with the IC50 value in all six cell types (Fig. 1a). The active form of PAK1 (phosphorylated PAK1 at Serine 144, pS144-PAK1, and Threonine 423, pT423-PAK1) was examined by western blotting using their specific antibodies. The expression levels of pS144-PAK1 and pT423-PAK1 seemed to support the correlation between PAK1 level and IC50 value in these NSCLC cell types (Fig. 1a).

We then transfected higher PAK1-expressing H441 and H23 cells with shPAK1, and we overexpressed PAK1, via its expression vector, in the lower PAK1-expressing H358 and H1355 cells. Western blotting showed the expected decrease in PAK1 expression by shPAK1 transfection and the expected increase by PAK1 overexpression (Fig. 1b upper panel). The cisplatin IC50 value decreased in the PAK1-knockdown H441 and H23 cells, and it increased in the PAK1-overexpressing H358 and H1355 cells when compared with their control cells (Fig. 1b lower panel). These results suggest that PAK1 may contribute to the cisplatin resistance observed in NSCLC cells.

### MEK/ERK signaling plays a more important role than PI3K/AKT signaling in PAK1-mediated cisplatin resistance

We examined whether MEK/ERK signaling could make a greater contribution than PI3K/AKT signaling to PAK1-mediated cisplatin resistance. PAK1-overexpressing H358 and H1355 cells were treated with the AKT inhibitor perifosine or the MEK/ERK inhibitor AZD6244. The expression of pAKT and pERK were modulated as expected by perifosine and AZD6244 (Fig. 1c upper panel). The MTT assay indicated that the cell viability in the high-PAK1 expressing cell types was more markedly decreased by AZD6244 than by perifosine (Fig. 1c lower panel). The cell viability in the PAK1-overexpressing cells was almost completely restored by AZD6244 treatment when compared with their control cells (Fig. 1c lower panel). These results suggest that MEK/ERK signaling may play a more important role than PI3K/AKT signaling in PAK1-mediated cisplatin resistance.

### An increase in β-catenin expression is responsible for PAK1-mediated cisplatin resistance via GSK3β phosphorylation at Serine 9 triggered by ERK signaling

We examined whether PAK1-induced ERK activation could increase β-catenin protein stability due to GSK3β phosphorylation at Serine 9 and whether an increase in β-catenin expression may be responsible for PAK1-mediated cisplatin resistance. PAK1-overexpressing H1355 and H23 cells were treated with AZD6244 and/or transfected to express ectopic β-catenin. Western blotting showed that β-catenin expression was markedly increased by PAK1 overexpression, but the increase in PAK1 expression was rescued by AZD6244 treatment in both PAK1-overexpressing cell types (Fig. 1d upper panel). The increase in β-catenin due to PAK1 overexpression in H1355 and H23 cells occurred via an increase in GSK3β phosphorylation at Serine 9, which increased the β-catenin protein stability. This increased stability due to GSK3β phosphorylation was almost completely rescued by AZD6244 plus MG132 treatment (Fig. 1e). The MTT assay indicated that the increase in cell viability by PAK1 overexpression was almost completely suppressed by AZD6244 treatment. However, the decrease in cell viability by AZD6244 in both cell types was almost completely rescued by ectopic β-catenin expression (Fig. 1d lower panel). These results suggest that an increase in β-catenin due to GSK3β phosphorylation at Serine 9 may be responsible for PAK1-mediated cisplatin resistance.

### β-catenin-induced stemness is responsible for PAK1-mediated cisplatin resistance

**Figure 2:**
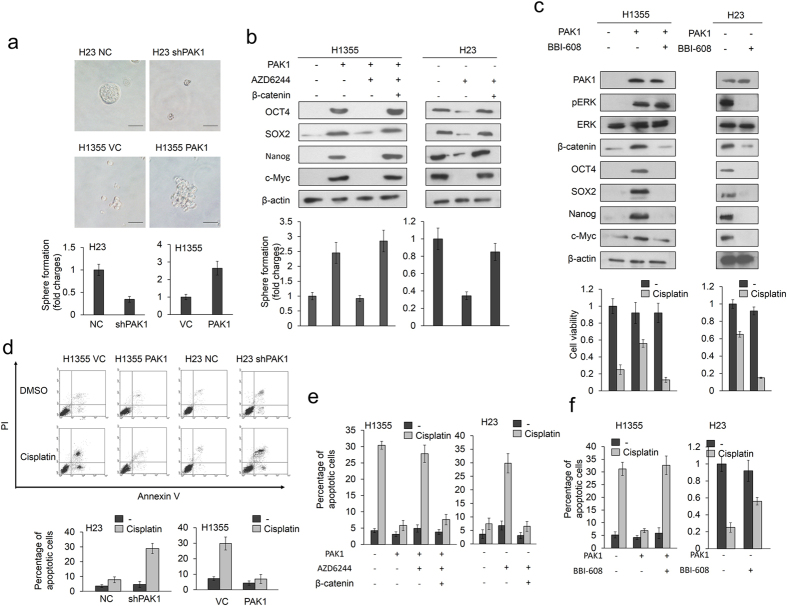
β-catenin-induced stemness is responsible for PAK1-mediated cisplatin resistance. (**a**) Sphere formation abilities in H23 and PAK1-overexpressing H1355 cells were identified in a low-attachment plate by incubation in serum-free media for 1 week. The photographs show sphere colonies in H23 and PAK1-overexpressing H1355 cells (upper panel). The graphs show the average number of spheres in triplicate samples (lower panel). (**b**) H23 and PAK1-overexpressing H1355 cells were transfected with a β-catenin overexpression plasmid for 24 h, followed by treatment with the MEK/ERK inhibitor AZD6244 for 5 h. The cells were then incubated for 1 week in a low-attachment plate in serum-free media and evaluated for sphere formation abilities. Expression of the cancer stem cell markers OCT4, SOX2, Nanog, and cmyc was identified using western blotting. (**c**) H23 and PAK1-overexpressing H1355 cells were treated with a cell stemness inhibitor (10 μM BBI-608) for 5 h. The inhibitor was then removed and the cells were treated with 25 μM cisplatin for an additional 48 h. Cell viability was then evaluated by MTT assay. (**d**) H1355 and H23 cells were transfected with PAK1 expression vector and shPAK1 for 24 h. The cells were then treated with 0.1% DMSO or 25 μM cisplatin for 24 h and subjected to annexin-V and PI staining, followed by a flow cytometry analysis. The percentages of apoptotic cells in the annexin V+/PI- and the annexin-V+/PI+ populations were determined. (**e**) H23 and PAK1-overexpressing H1355 cells were transfected with β-catenin overexpression plasmid for 24 h, followed by treatment with AZD6244 for 5 h. The inhibitor was then removed and the cells were treated with cisplatin for an additional 48 h. (**f**) H23 and PAK1-overexpressing H1355 cells were treated with a cell stemness inhibitor (10 μM BBI-608) for 5 h. The inhibitor was then removed and the cells were treated with 25 μM cisplatin for an additional 48 h. Cell apoptosis was evaluated by flow cytometry. All experiments were performed three independent times. The mean values and the standard deviations are indicated as columns with error *bars*. The samples were derived from the same experiment and gels/blots were processed in parallel. Full-length blots are presented in Supplementary Figure S3.

Cell stemness is associated with β-catenin activation and cancer drug resistance^13^,^14^,^15^,^16^,^17^. We examined the possibility that an increase in β-catenin expression triggered by PAK1 could promote cisplatin resistance by enhancing cell stemness. The sphere cell formation assay indicated a marked increase and decrease in sphere formation following PAK1 manipulation in H23 and H1355 cells (Fig. 2a upper panel). Representative sphere cells are shown in Fig. 2a (upper panel). Western blotting indicated that the expression of the stemness markers OCT4, SOX2, Nanog, and c-Myc was markedly increased by PAK1 overexpression in H1355 cells, but the increase in these four proteins by PAK1 overexpression was suppressed by AZD6244 treatment; interestingly, the four proteins were re-expressed in PAK1-overexpressing H1355 cells treated with AZD6244 and expressing ectopic β-catenin (Fig. 2b left panel). A similar response was observed in H23 cells subjected to AZD6244 and/or ectopic β-catenin expression (Fig. 2b right panel). The efficacy of sphere cell formation was increased by PAK1 overexpression, but was decreased by AZD6244 treatment in PAK1-overexpressing H1355 cells. However, the sphere cell formation was almost completely rescued in PAK1-overexpressing H1355 cells treated with AZD6244 but also expressing ectopic β-catenin and in parental H23 cells (Fig. 2b lower panel).

High levels of CD44 and low levels of CD24 induce stem-like activities in breast cancer cells^18^. Therefore, the CD44+/CD24− stem cell profiles of PAK1-overexpressing H1355 and PAK1-knockdown H23 cells were determined by flow cytometry. The representative CD44+/CD24− cell distributions modulated by PAK1 manipulation in H23 and H1355 cells are shown in Supplementary Figure 1a (upper panel). The percentage of CD44+/CD24- cells was decreased and increased by PAK1 manipulation in H23 and H1355 cells, respectively (Supplementary Figure 1a lower panel). We examined the possibility that the percentage of CD44+/CD24- cells modulated by PAK1 manipulation could be changed by MEK/ERK inhibitor treatment and/or ectopic β-catenin expression. The percentage of CD44+/CD24- cells was markedly increased by PAK1 overexpression in H1355 cells but rescued by AZD6244 treatment; however, this restoration of the percentage of CD44+/CD24- cells was nearly completely reversed by ectopic β-catenin expression (Supplementary Figure 1b left panel). Similar findings were also observed in H23 cells subjected to the same treatments. Moreover, the increase in the percentage of CD44+/CD24− cells by PAK1 was rescued by the stemness inhibitor BBI-608 treatment in both cell types (Supplementary Figure 1c).

We next examined whether BBI-608 treatment could overcome PAK1-mediated cisplatin resistance. Western blotting indicated that the increases in β-catenin, OCT4, SOX2, Nanog, and c-Myc expression in PAK1-overexpressing H1355 cells were almost completely eliminated by BBI-608 treatment. A similar finding in these five protein expressions was also observed in H23 cells subjected to BBI-608 treatment (Fig. 2c upper panel). However, the expression of PAK1 and pERK was nearly unchanged by BBI-608 treatment in H23 and PAK1-overexpressing H1355 cells. We therefore suggest that PAK1-mediated cisplatin resistance overcome by BBI-608 treatment may be through modulating β-catenin-induced stemness.

The MTT assay showed that the increases in cisplatin-induced cell viability due to PAK1 overexpression in H1355 cells and parental H23 cells was almost completely suppressed by BBI-608 treatment (Fig. 2c lower panel). The Annexin V-PI staining analysis further confirmed that PAK1-mediated cisplatin resistance operates through apoptosis (Fig. 2d). The percentage of apoptotic cells induced by cisplatin was markedly decreased by PAK1 overexpression in H1355 cells and parental H23 cells when compared with their control cells (Fig. 2e). However, the decrease in the percentage of apoptotic cells by cisplatin in cells ectopically expressing PAK1 was increased markedly by AZD6244 treatment, and further restored by AZD6244 plus ectopic β-catenin expression in PAK1-overexpressing H1355 cells and parental H23 cells (Fig. 2e). The percentage of apoptotic cells induced by cisplatin was markedly decreased by PAK1 overexpression in H1355 cells, but the increase in the percentage of apoptotic cells due to PAK1 overexpression was almost completely restored by BBI-608 treatment (Fig. 2f left panel). Similarly, the percentage of apoptotic cells induced by cisplatin was markedly increased by BBI-608 treatment in parental H23 cells (Fig. 2f right panel). These results suggest that an increase in β-catenin expression triggered by PAK1 may induce cell stemness and, in turn, confer cisplatin resistance.

### PAK1 is positively correlated with pERK and β-catenin expression and is associated with an unfavorable response to cisplatin-based chemotherapy and with poor outcomes

**Figure 3:**
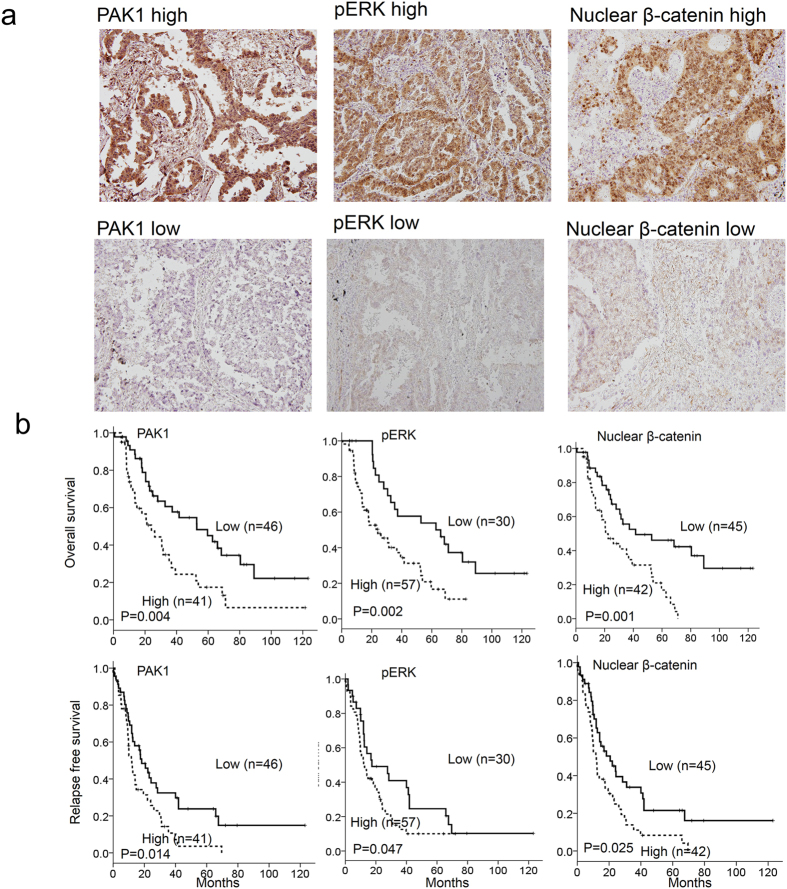
High-PAK1, high-pERK, and high-nuclear β-catenin expressions are associated with poor OS and RFS in NSCLC. (**a**) A representative figure of PAK1, pERK, and nuclear β-catenin expression in lung cancer patients. (**b**) Lung cancer patients with tumors showing high-PAK1, high-pERK, and high-nuclear β-catenin expression had poor outcomes.

Eighty-seven tumors from NSCLC patients were enrolled to examine whether PAK1 could be associated with pERK and β-catenin expression. The expression of PAK1, pERK, and β-catenin in lung tumors was evaluated by immunohistochemistry. Representative immunostaining results are shown in Fig. 3 (upper panel). High-pERK and high-nuclear β-catenin expression occurred more frequently in high-PAK1 tumors than in low PAK1 tumors (78% vs. 56%, P = 0.040 for pERK; 61% vs. 37%, P = 0.025 for β-catenin; Table 1). A positive correlation was observed between pERK and nuclear β-catenin in lung tumors (58% vs. 30.0%, P = 0.013; Table 1).

The entire study population had undergone cisplatin-based chemotherapy. The responses to cisplatin-based chemotherapy were collected from chart reviews for retrospective study. As shown in Table 2, an unfavorable response to cisplatin-based chemotherapy more commonly occurred in patients with high-PAK1, high-pERK, or high-nuclear β-catenin tumors than in those with low-PAK1, low-pERK, or negative nuclear β-catenin tumors (63% vs. 37% P = 0.014 for PAK1, 65% vs. 20% P < 0.001 for pERK, 67% vs. 33% P = 0.002 for β-catenin; Table 2).

Kaplan-Meier analysis indicated that a shorter overall survival (OS) and relapse free survival (RFS) occurred in high-PAK1, high-pERK, and high-nuclear β-catenin tumors than in their counterparts (Fig. 3 lower panel). Cox regression analysis further indicated that the prognostic significance of high-PAK1, high-pERK, high-nuclear β-catenin, high-PAK1/high-pERK, and high-ERK/high-nuclear β-catenin tumors for OS and RFS, compared with their counterparts, was still observed in this study population (Table 3). These results strongly support the mechanism indicated by the cell models, whereby PAK1 confers cisplatin resistance and poor outcomes via β-catenin-mediated stemness.

## Discussion

NSCLC is highly heterogeneous and resistant to available therapeutic agents, with a five year survival rate of less than 15%. The basis of NSCLC heterogeneity and drug resistance has been difficult to determine, although genetic alterations and aberrations in signaling resistance have been reported^19^. Hypoxia-induced cell stemness leads to drug resistance and poor prognosis in lung adenocarcinoma^20^. Consistently, tumor malignancy and drug resistance may be explained in part by cancer stemness^19^. In the present study, we have provided evidence that β-catenin-mediated stemness may be responsible for PAK1-induced cisplatin resistance and poor outcomes in NSCLC.

PAK1 directly phosphorylates β-catenin proteins at Serine 675 and this leads to a more stable and transcriptionally activated β-catenin in colon cancer cells^3^. However, in the present study, the increase in β-catenin protein stability and its transcriptional activation occurred through GSK3β inactivation by Serine 9 phosphorylation due to PAK1-activated ERK signaling (Figs 1 and 2). β-catenin signaling is responsible for cell stemness in osteosarcoma and hepatocellular carcinoma. However, the role of the β-catenin signaling mediated by PAK1 in lung cancer cell stemness is not fully understood, although the PAK1-mediated stem-like phenotype confers sunitinib resistance via NF-κB/IL-6 activation in renal cell carcinoma^9^. Therefore, the present study is the first to demonstrate that PAK1 confers cisplatin resistance in NSCLC via β-catenin-mediated stemness.

A link between EMT and cancer stemness has been demonstrated by accumulated evidence^21^,^22^,^23^,^24^. The cancer stem cells concept implicates to predict the propensity of tumors to metastasize^24^,^25^,^26^,^27^. We therefore examined the possibility that PAK1 could promote cell invasion via the pERK/β-catenin axis. A marked increase and decrease in invasion ability was observed in H23 and H1355 cells following PAK1 manipulation (Supplementary Figure 2a right panel). Representative invasive cells of both cell types in matrigel membranes are shown in Supplementary Figure 2a (Left panel). The invasion ability in H1355 cells was increased by PAK1 overexpression, but was decreased by AZD6244 treatment. However, the invasion ability was almost completely rescued in PAK1-overexpressing H1355 cells with AZD6244 treatment and in H23 cells with ectopic β-catenin expression (Supplementary Figure 2b). The increase of invasion ability by PAK1 overexpression in H1355 cells and in parental H23 cells was almost completely eliminated by BBI-608 treatment (Fig. 2c lower panel). We therefore suggest that PAK1 may promote cell invasion via the pERK/β-catenin axis-mediated stemness.

In summary, we provided evidence that PAK1 confers cisplatin resistance in NSCLC cells by increasing β-catenin expression and stemness due to ERK-mediated GSK3β inactivation. The expression of PAK1, pERK, and β-catenin was associated with an unfavorable response to cisplatin-based chemotherapy and poor outcomes in NSCLC patients. Therefore, we suggest that combining an MEK/ERK inhibitor such as AZD6244 with cisplatin in the clinical setting might improve cisplatin-based chemotherapy sensitivity and outcomes in NSCLC patients who harbor high-PAK1 tumors.

## Materials and Methods

### Study subjects

The study included 87 patients who underwent resected at the Department of Chest Surgery, Taichung Veteran General Hospital, Taichung, Taiwan, between June 1994 and December 2006. The tumor type and stage of each specimen was histologically determined according to the World Health Organization’s (WHO) classification system. The present experiments were conducted in accordance with the Declaration of Helsinki^28^. The IRB protocol 201305018 was approved by Taipei Medical University Hospital and written informed consent was obtained from the patients prior to study enrollment.

### Tumor response

Among these patients, 87 patients were treated with cisplatin-based chemotherapy. Responses were categorized as follows: Complete response (CR): a complete disappearance of all the tumors; partial response (PR): a decrease in size or number of the tumor lesions by 50% or more; progressive disease (PD): at least 25% increase in size or number of the tumor lesions; and stable disease (SD): neither sufficient shrinkage to qualify for partial response nor sufficient increase to qualify for progressive disease. Therefore, the favorable response (CR and PR) is a decrease in tumor size of least 50% or more.

### Cell lines

A549, H441, H23, H358 and H1355 cells were obtained from the American Type Culture Collection (ATCC) and cultured as described. CL1-5 cells were kindly provided by Dr. P.-C. Yang (Department of Internal Medicine, National Taiwan University Hospital, Taiwan). These cells were cultured and stored according to the suppliers’ instructions and used at passages 5 to 20. Once resuscitated, cell lines are routinely authenticated (once every 6 months, cells were last tested in June 2015) through cell morphology, proliferation rate, a panel of genetic markers, and contamination checks.

### Chemicals and antibodies

Cisplatin, Perifosine and AZD6244 was obtained from Selleckchem.com (Houston, TX). BBI-608 was obtained from BioVision (Mountain View, CA). All other chemicals were acquired from Sigma Chemical (St. Louis, MO) unless otherwise indicated. Anti-total AKT, anti–phospho-AKT (pAKT), anti-total ERK, anti–phospho-ERK (pERK) and anti-phospho (Ser33, Ser 37, Thr 41)-β-catenin (p-β-catenin) antibodies were obtained from Cell Signaling (Danvers, MA). Anti-PAK1, anti–phospho-PAK1 (pS144-PAK1), anti-total GSK3β, anti–phospho- GSK3β (pS9-GSK3β), anti-SOX2 and anti-OCT4 were obtained from Genetex (Irvine CA). Anti-phospho-PAK1 (pT423-PAK1) antibody was obtained from Abnova (Taiwan). All other antibodies were purchased from Santa Cruz Biotechnology (Dallas, TX).

### Plasmid constructs and transfection

The PAK1-overexpression plasmid was purchased from Origene (Rockville, MD). The β-catenin-overexpression plasmid was purchased from Addgene (Addgene Company, Cambridge). PAK1 shRNA (TRCN0000352635) was purchased from National RNAi Core Facility, Academia Sinica, Taiwan. Different concentrations of expression plasmids were transiently transfected into lung cancer cells (1 × 10^6^) using the Turbofect reagent (Formentas, USA). After 48 h, cells were harvested and whole cell extracts were assayed in subsequent experiments.

### Immunohistochemical analysis

The immunohistochemical procedures and quantification methods were as described previously^29^.

### MTT cytotoxicity assay

The cell lines were cultured in a humidified incubator containing 95% air and 5% CO2 at 37 °C in 96-well flat-bottomed microtiter plates containing RPMI and DMEM supplemented with 10% heat-inactivated fetal bovine serum (FBS), 100 U/ml penicillin, and 100 U/ml streptomycin. Before cisplatin treatment, the cells in the exponential growth phase were pretreated with overexpression and knockdown plasmids for 24 h or procaine for 2 h. After 48 h of incubation, the *in vitro* cytotoxic effects of these treatments were determined by MTT assay (at 570 nm) and the cell viability was expressed as a percentage of the control (untreated) cells (% of control).

### Annexin-V staining

The cells were collected by trypsinization and centrifugation at 1,000 *g* for 5 min. Following resuspension in binding buffer (10 mM HEPES-NaOH, 140 mM NaCl, 2.5 mM CaCl_2_) at a final cell density of 1–2 × 10^6^ cells/ml, 100 μl of a single-cell suspension (1–2 × 10^5^ cells) was incubated with 5 μl annexin V-FITC and 5 μl PI for 15 min at room temperature in the dark. After addition of 400 μl of binding buffer, the samples were analyzed by using a BD FACSCalibur flow cytometer (BD Biosciences, San Jose, CA) within 1 h. For each sample, 10,000 events were counted.

### Boyden chamber assay

Cell invasion ability was evaluated by Boyden chamber assay using a Falcon^®^ cell culture insert for 24-well plate with 8.0 μm pore transparent PET membrane (BD Biosciences). Cells (1 × 10^5^) were plated onto the upper compartment of chamber coated with Geltrex^®^. The lower compartment was filled with complete medium. The chamber was incubated at 37 °C for 16 h. Invasive cells on the bottom of inserts were fixed with 100% ethanol, stained with Giemsa and observed under a microscope. The number of invasive cells was counted and averaged in three random microscopic fields for each sample group. All experiments were performed in three independent times.

### Sphere culture

Single cell suspensions of indicated cells (5000 cells/well) were cultured on ultralow attachment plates (Corning, Lowell, MA). Cells were grown in a serum-free growth medium supplemented with EGF and FGF. After 10 days of incubation, percentage of sphere forming cells were calculated by dividing the number of spheres by the number of cells seeded. Spheres >50 μm diameter were counted after 10 days.

### Flow cytometry

Cells were washed once with phosphate-buffered saline (PBS) and then harvested with 0.05% trypsin/0.025% EDTA. Detached cells were washed with PBS containing 1% FCS and 1% penicillin/streptomycin (wash buffer), and resuspended in the wash buffer (10^6^ cells/100 μl). Combinations of fluorochrome-conjugated monoclonal antibodies obtained from BD Biosciences (San Diego, CA, USA) against human CD44 (PE-Cyanine5; cat. #15–0441) and CD24 (PE; cat. #15–0247) or their respective isotype controls were added to the cell suspension at concentrations recommended by the manufacturer and incubated at 4 °C in the dark for 30 to 40 min. The labeled cells were washed in the wash buffer, then fixed in PBS containing 1% paraformaldehyde, and then analyzed on BD FACSCalibur flow cytometer.

### Statistical analysis

Statistical analysis was performed using the SPSS statistical software program (Version 18.0; SPSS Inc., USA). The association between tumor response and PAK1 protein expression was analyzed by the chi-square test. Survival plots were generated using the Kaplan-Meier method, and differences between patient groups were determined by the log-rank test. Multivariate Cox regression analysis was performed to determine OS and RFS. The analysis was stratified for all known variables (age, gender, smoking status, tumor type, and tumor stage) and protein expression.

## Additional Information

**How to cite this article**: Chen, M.-J. *et al*. PAK1 confers chemoresistance and poor outcome in non-small cell lung cancer via β-catenin-mediated stemness. *Sci. Rep*. **6**, 34933; doi: 10.1038/srep34933 (2016).

## Supplementary Material

Supplementary Information

## Figures and Tables

**Table 1:** The correlation of PAK1 with pERK and nuclear β-catenin expression and their each correlation in lung cancer patients.

	No	pERK			Nuclear β-catenin		
		Low (%)	High (%)	P	Low (%)	High (%)	P
**PAK1**							
Low	46	20(44)	26(56)	0.040	29(63)	17(37)	0.025
High	41	9(23)	31(78)		16(39)	25(61)	
**pERK**							
Low	30				21(70)	9(30)	0.013
High	57				24(42)	33(58)	

**Table 2:** The association of PAK1, pERK and nuclear β-catenin expression with the response to cisplatin-based therapy in lung cancer patients.

	No	Tumor response		
		Poor	Favorable	P
**PAK1**				
Low	46	17(37)	29(63)	0.014
High	41	26(63)	15(36)	
**pERK**				
Low	30	6(20)	24(80)	<0.001
High	57	37(65)	20(35)	
**Nuclear β-catenin**				
Low	45	15(33)	30(67)	0.002
High	42	28(67)	14(33)	

Complete response (CR): a complete disappearance of all the tumors; partial response (PR): a decrease in size or number of the tumor lesions by 50% or more; progressive disease (PD): at least 25% increase in size or number of the tumor lesions; and stable disease (SD): neither sufficient shrinkage to qualify for partial response nor sufficient increase to qualify for progressive disease. Therefore, the favorable response (CR and PR) is the decrease of tumor size at least 50% or more.

**Table 3:** Cox regression analysis for the prognostic value of PAK1, pERK and nuclear β-catenin on OS and RFS in lung cancer patients.

	OS				RFS			
	Case No.	HR*	95% CI	P	Case No.	HR*	95% CI	P
**PAK1**								
Low	46	1			46	1		
High	41	2.22	1.28–3.85	0.004	41	1.69	1.04–2.73	0.033
**pERK**								
Low	30	1			30	1		
High	57	2.94	1.53–5.63	0.001	57	1.77	1.02–3.09	0.043
**Nuclear β-catenin**								
Low	45	1			45	1		
High	42	2.29	1.26–4.16	0.007	42	1.84	1.12–3.04	0.017
**PAK1/pERK**								
Low/Low	20	1			20	1		
High/Low	10	0.95	0.33–2.79	0.928	10	1.43	0.57–3.56	0.442
Low/High	26	1.74	0.76–4.02	0.193	26	1.53	0.75–3.14	0.244
High/High	31	4.72	2.18–10.2	<0.001	31	2.42	1.23–4.76	0.011
**pERK/ nuclear β-catenin**								
Low/Low	29	1			29	1		
High/Low	16	2.06	0.83–5.12	0.12	16	1.75	0.78–3.91	0.174
Low/High	17	2.00	0.78–5.12	0.149	17	1.95	0.87–4.40	0.107
High/High	25	3.29	1.61–6.72	0.001	25	2.39	1.28–4.45	0.006

OS: overall survival; HR: Hazard ratio; RFS: relapse free survival.

*HR for all cases was adjusted by age, gender, smoking status and stage.
