# Corrigendum: PAK1 confers chemoresistance and poor outcome in non-small cell lung cancer via β-catenin-mediated stemness

**DOI:** 10.1038/srep38463

**Published:** 2016-12-09

**Authors:** Ming-Jenn Chen, De-Wei Wu, Yao-Chen Wang, Chi-Yi Chen, Huei Lee

Scientific Reports 6: Article number: 3493310.1038/srep34933; published online: 10 07 2016; updated: 12 09 2016

In this Article, an affiliation for Ming-Jenn Chen was omitted. The correct affiliations for Ming-Jenn Chen are listed below:

Department of Surgery, Chi Mei Medical Center, Tainan, Taiwan.

Department of Sports Management, College of Leisure and Recreation Management, Chia Nan University of Pharmacy and Science, Tainan, Taiwan.

