# Author Correction: PAK1 confers chemoresistance and poor outcome in non-small cell lung cancer via β-catenin-mediated stemness

**DOI:** 10.1038/s41598-020-74206-6

**Published:** 2020-10-23

**Authors:** Ming-Jenn Chen, De-Wei Wu, Yao-Chen Wang, Chi-Yi Chen, Huei Lee

**Affiliations:** 1 Department of Surgery, Chi Mei Medical Center, Tainan, Taiwan; 2 Graduate Institute of Cancer Biology and Drug Discovery, Taipei Medical University, Taipei, Taiwan; 3 School of Medicine, Chung Shan Medical University, Taichung, Taiwan; 4 Division of Pulmonary Medicine, Department of Internal Medicine, Chung Shan Medical University Hospital, Taichung, Taiwan; 5 Department of Surgery, Chung Shan Medical University, Taichung, Taiwan; 6 Department of Sports Management, College of Leisure and Recreation Management, Chia Nan University of Pharmacy and Science, Tainan, Taiwan

Correction to: *Scientific Reports* 10.1038/srep34933, published online on 07 October 2016

This Article contains an error in the affiliation for Yao-Chen Wang, which is incorrectly given as ‘Department of Internal Medicine, Chung Shan Medical University, Taiwan’. The correct affiliations are listed below:

School of Medicine, Chung Shan Medical University, Taichung, Taiwan.

Division of Pulmonary Medicine, Department of Internal Medicine, Chung Shan Medical University Hospital, Taichung, Taiwan.

