# Supplementary material for: PAK1 confers chemoresistance and poor outcome in non-small cell lung cancer via β-catenin-mediated stemness
Source: Sci Rep. 2016 Oct 7;6:34933. doi: 10.1038/srep34933 (PMC5054675; doi:10.1038/srep34933)
Supplement: Supplementary Information [file srep34933-s1.doc]

**PAK1 confers chemoresistance and poor outcome in non-small cell lung cancer via β-catenin-mediated stemness**

Ming-Jenn Chen^2^, De-Wei Wu^1^, Yao-Chen Wang3, Chi-Yi Chen^4^, and Huei Lee^1^

**Supplementary Figure legend**

**Supplementary Figure S1. A CD44+/CD24-/low subpopulation is evident in PAK1-overexpressing lung cancer cells.** (a) H1355 and H23 cells were transfected with PAK1 expression vector and shPAK1 for 24 h. The cells were subjected to CD44 and CD24 staining, followed by a flow cytometry analysis. (b) H23 and PAK1-overexpressing H1355 cells were transfected with β-catenin overexpression plasmid for 24 h, followed by treatment with AZD6244 for 5 h. (c) H23 and PAK1-overexpressing H1355 cells were treated with a cell stemness inhibitor (10 μM BBI-608) for 5 h. CD44+/CD24-/low subpopulation was evaluated by flow cytometry*.*

**Supplementary Figure S2. PAK1 promotes cell invasion via stemness by the pERK/β-catenin axis.** (a) The invasion ability in H23 and PAK1-overexpressing H1355 cells were evaluated by Boyden chamber for 16 h. The representative invasive cells of H23 and PAK1-overexpressing H1355 cells are shown on membranes. The graphs show the relative of invasion abilities in H23 and PAK1-overexpressing H1355 cells as compared its counterpart. (b) H23 and PAK1-overexpressing H1355 cells were transfected with a β-catenin overexpression plasmid for 24 h, followed by treatment with the ERK inhibitor AZD6244 for 5 h. The invasion ability in H23 and PAK1-overexpressing H1355 cells was evaluated by Boyden chamber for 16 h. (c) H23 and PAK1-overexpressing H1355 cells were treated with a cell stemness inhibitor (10 μM BBI608) for 5 h. The inhibitor was then removed to treat with 25 μM cisplatin for an additional 48 h. The invasion ability in H23 and PAK1-overexpressing H1355 cells was evaluated by Boyden chamber for 16 h.

**Supplementary Figure S3.** Full-length blots for main figures corresponded to Fig. 1a, 1b, 1c and 1d.

**Supplementary Figure S4.** Full-length blots for main figures corresponded to Fig. 1e, 2b and 2c.

**Supplementary Figure S1.**

**
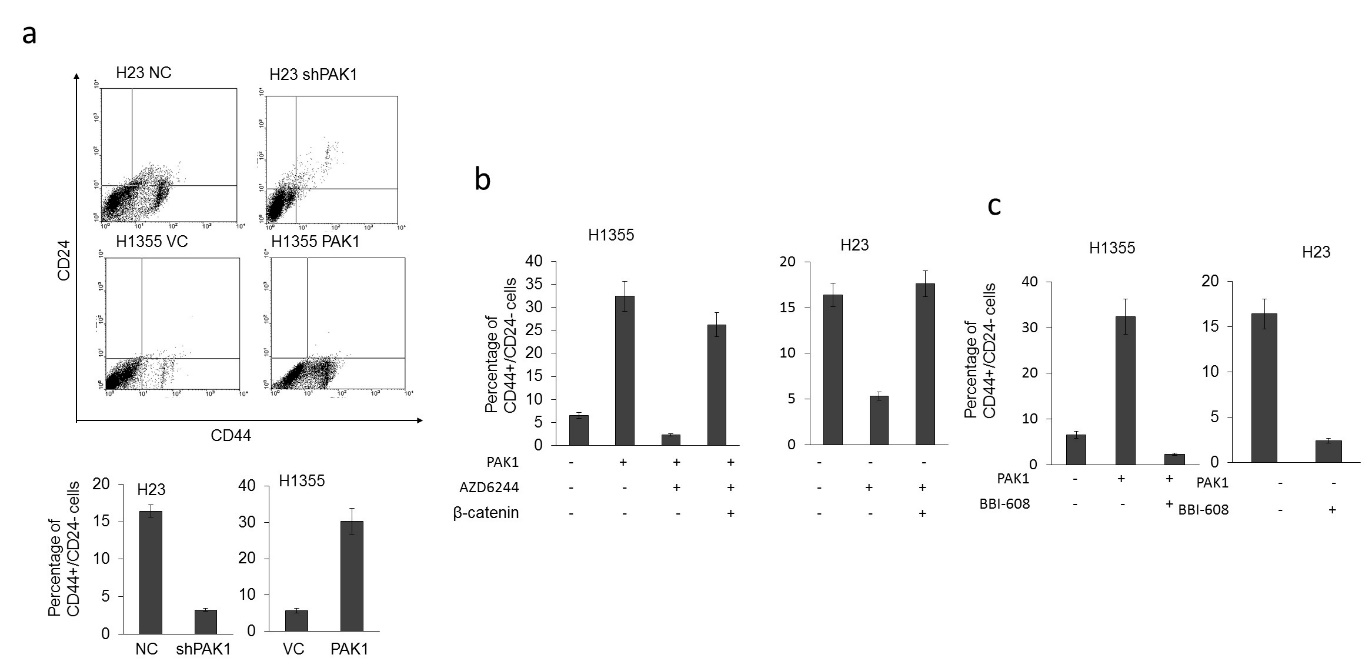
**

**Supplementary Figure S2.**

**
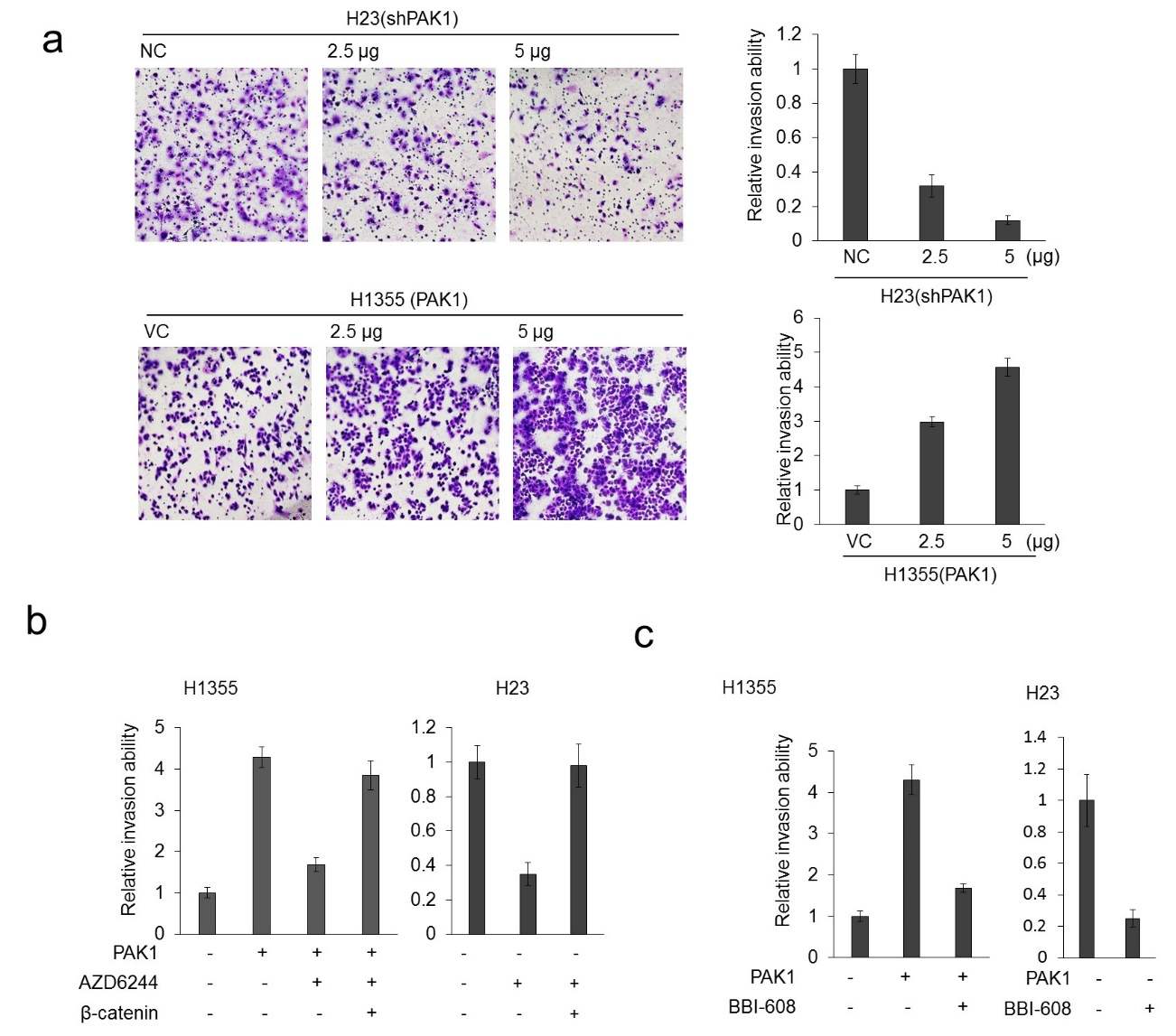
**

**Supplementary Figure S3.**

**
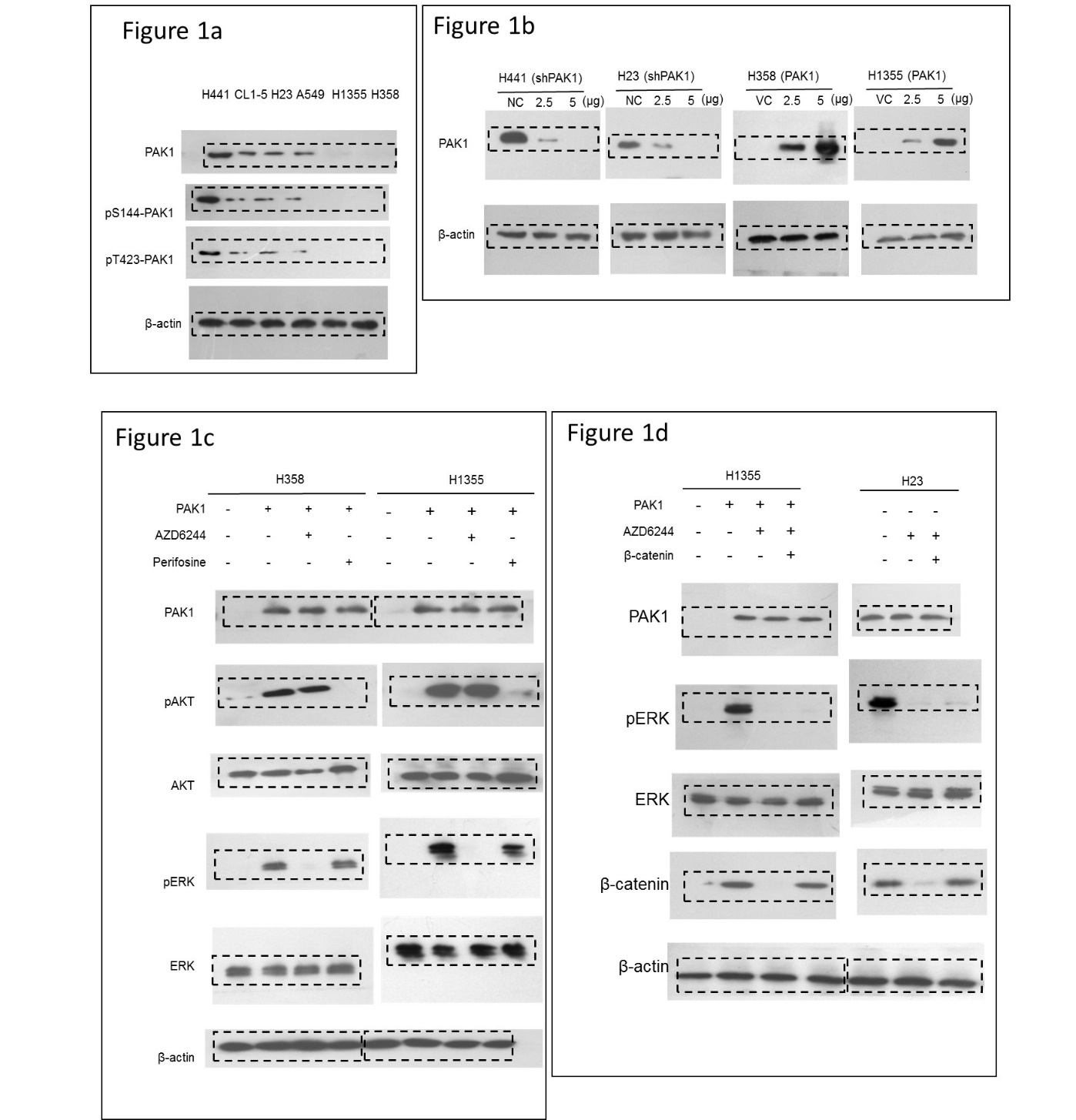
**

**Supplementary Figure S4.**

**
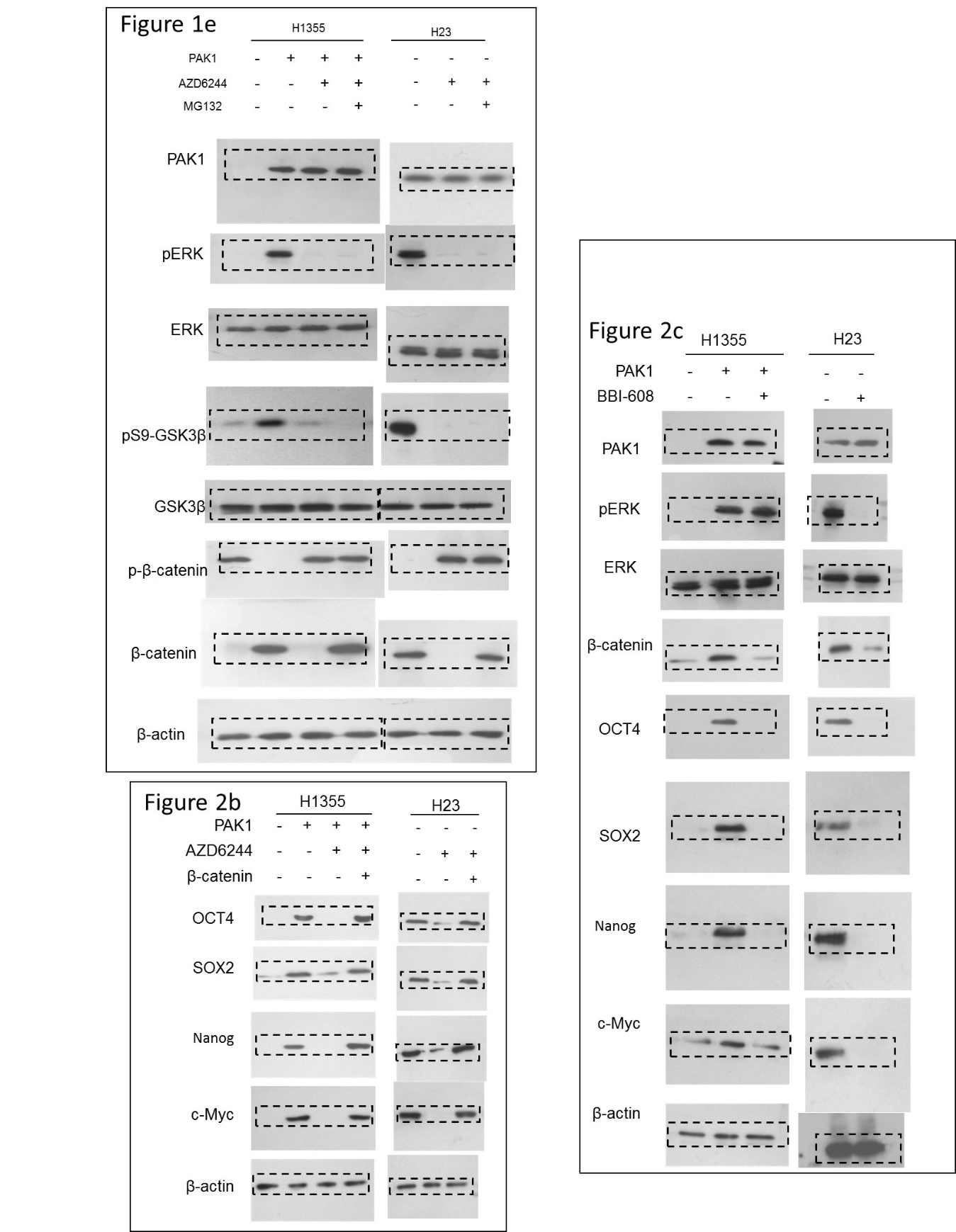
**
